# Shoulder Joint Hybrid Assistive Limb Treatment for Chronic Stroke Patients with Upper Limb Dysfunction

**DOI:** 10.3390/jcm12031215

**Published:** 2023-02-03

**Authors:** Masakazu Taketomi, Yukiyo Shimizu, Hideki Kadone, Shigeki Kubota, Yuta Kagai, Yoshitaka Okamoto, Yasushi Hada, Masashi Yamazaki

**Affiliations:** 1 Doctoral Program in Clinical Science, Graduate School of Comprehensive Human Science, University of Tsukuba, Tsukuba 305-8575, Japan; 2 Department of Rehabilitation Medicine, Faculty of Medicine, University of Tsukuba, Tsukuba 305-8575, Japan; 3 Center for Innovative Medicine and Engineering, University of Tsukuba Hospital, Tsukuba 305-8575, Japan; 4 Division of Regenerative Medicine for Musculoskeletal System, Faculty of Medicine, University of Tsukuba, Tsukuba 305-8575, Japan; 5 Department of Rehabilitation Medicine, University of Tsukuba Hospital, Tsukuba 305-8576, Japan; 6 Department of Orthopedic Surgery, Faculty of Medicine, University of Tsukuba, Tsukuba 305-8575, Japan

**Keywords:** hybrid assistive limb (HAL), shoulder, rehabilitation, robotic rehabilitation, stroke, upper limb impairment

## Abstract

Upper extremity dysfunction after stroke affects quality of life. Focusing on the shoulder joint, we investigated the safety and effectiveness of rehabilitation using a shoulder joint hybrid assistive limb (HAL). Eight patients with chronic stroke and upper extremity functional disability were enrolled and used a shoulder joint HAL, which assisted shoulder movement based on the user’s intention, through myoelectric activation of the shoulder flexor. Ten training sessions of 30–40 min each were performed to assist voluntary movement of upper limb elevation on the affected side through triggering the deltoid muscle. All patients completed the interventions without shoulder pain. Surface electromyography evaluation indicated post-intervention improvement in coordinated movement of the affected upper extremity. Significant improvements in voluntary and passive shoulder joint range of motion were obtained after the intervention, suggesting improvement in shoulder muscle strength. A significant decrease in the modified Ashworth scale and improvements in functional scores in the upper limb were also observed. Along with safe use for our study patients, the shoulder HAL provided appropriate motor learning benefits. Improvements in shoulder joint function and whole upper limb function were observed, suggesting that HAL could be an optimal treatment method.

## 1. Introduction

Upper extremity dysfunction due to stroke significantly affects activities of daily living, influencing quality of life [1]. It has been reported that approximately 50% of patients with stroke continue to experience upper limb dysfunction six months after stroke onset, and approximately 60% of those with severe or complete paralysis are unable to perform any movement with their affected limbs [2,3,4]. Recent advances in imaging examinations, such as functional magnetic resonance imaging and near-infrared spectroscopy, have shown that brain plasticity or reorganization can be expected after stroke. Recently, robotic rehabilitation has emerged as a training method to improve patients’ limb dysfunction post-stroke [5,6]. The hybrid assistive limb (HAL) is an exoskeletal robot that controls and assists movements based on bioelectrical activity generated through voluntary movements. By means of generating feedback to the central nervous system, it has been hypothesized that this device stimulates functional recovery through inducing plasticity in the impaired central nervous system [7]. There are four types of HAL, namely, lower limb, single joint (for elbow and knee joints), and lumbar types. One study that used a single joint HAL for the elbows reported improvements in upper limb motor function in patients with stroke [8]. Focusing on shoulder dysfunction after stroke, we previously conducted training using a shoulder joint HAL developed by our research group in patients with stroke. Furthermore, we published a case report showing that this training could be performed safely, while improving shoulder joint function and coordinated movements of the upper limb on the affected side [9]. Rehabilitation therapy focusing on the shoulder joint is extremely important, as improving shoulder joint function not only improves activities of daily living, such as changing clothes, but also ameliorates distal control of the upper extremity and prevents shoulder pain [10,11,12]. In this study, we aimed to determine the utility of shoulder joint HAL training applied in eight patients with chronic stroke and moderate-to-severe upper limb dysfunction.

## 2. Materials and Methods

### 2.1. Patients

Eight patients (six males, two females) were enrolled in this study. Patients’ clinical data are shown in Table 1. The mean patient age (± standard deviation) was 68.4 ± 8.38 (range, 53–84) years. The mean time from stroke onset was 5.86 ± 6.40 (range, 0.93–19.7) years. All patients showed moderate-to-severe hemiplegia with a shoulder flexion manual muscle test (MMT) score of ≤2. Their grip power was <50% on the affected side compared with the unaffected upper limb and three patients were unable to complete grip dynamometer measurements. In Patient 8, bilateral grip power measurements could not be measured as the unaffected upper limb had been amputated at the hand level due to trauma in childhood. This study was conducted in accordance with the Declaration of Helsinki, with approval from the Ethics Committee of the Tsukuba University Faculty of Medicine (approval no.: TCRB18-38). All patients provided written informed consent for participation and publication, including the use of any accompanying images.

### 2.2. HAL Intervention

**Figure 1:**
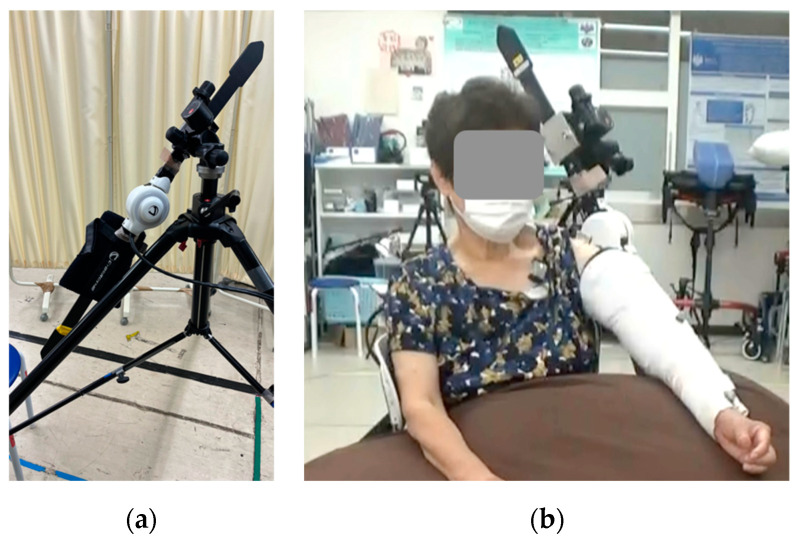
Images of the shoulder joint HAL device. (**a**) Single-joint HAL fixed to a tripod with attachments, (**b**) shoulder joint HAL fixed with splints and an elastic bandage. HAL, hybrid assistive limb.

We set up the single-joint HAL in accordance with previous studies [5,6]. In brief, the proximal section of the HAL was fixed to a tripod using an attachment, and the distal section was fitted to the patient’s upper arm with a belt for the elbow joint, which was attached to the HAL (Figure 1a). The elbow was extended to its full range of motion (ROM); the forearm was placed in a slightly externally rotated position to prevent external rotation of the humerus and was immobilized near the wrist joint using a splint and bandage (Figure 1b). Flexion electrodes were placed on the skin of the anterior deltoid fibers and triggered for upper limb elevation. Instead of using the electrodes as triggers, relaxation of the shoulder flexor muscle and gravity functioned to trigger extension. The ground was placed on the bone touching the site where the bone was palpable, without interfering with the surface electromyography device.

The upper limb raising angle during training was measured prior to fitting the HAL, and the shoulder joint ROM was initiated at approximately 20° less than the ROM at the shoulder joint and then gradually increased while observing the training condition. Two methods were used to adjust the actual angle: (i) adjustment of the HAL, and (ii) adjustment using a tripod attachment. The HAL angle could be adjusted from 0° to 120°, and was used for adjustment. When the HAL assist angle of 120° was considered to be insufficient, a further increase in the angle of elevation was obtained through changing the tilt of the HAL itself using a tripod attachment.

All eight patients who participated in the study underwent a total of 10 HAL training sessions, with each session lasting 30–40 min, with at least one week between each intervention. The actual training time for upper extremity raising was approximately 20–30 min, including breaks, after approximately 5–20 min for electrode preparation and HAL placement and removal.

**Figure 2:**
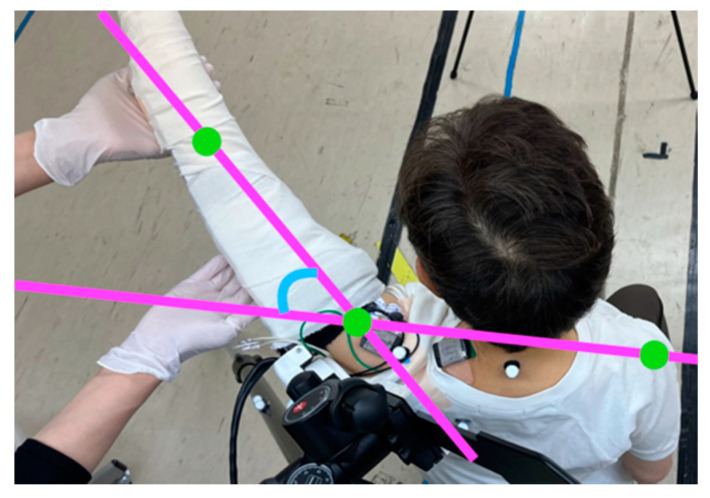
Images showing scapular elevation.

During training, a therapist stabilized the medial side of the patients’ forearms to avoid excessive internal rotation or flexion of the upper limbs during the raising of the upper limbs. The direction of upper limb elevation was evaluated while observing the raising of the scapular plane, which needs to be considered to prevent excessive interference between the humerus and scapula (Figure 2). The pace of each exercise was set so that patients could fully extend their arms one at a time to avoid vigorous raising and then repeat raising the upper arm.

Upper extremity elevation at a 30–45° angle between the line connecting the bilateral acromion and the line connecting the acromion to the elbow joint is optimal to avoid interference between the scapula and humerus.

### 2.3. Assessments

#### 2.3.1. Safety

Medical interviews and situational examinations were conducted to assess all participants for adverse symptoms, such as shoulder pain, occurrence of additional physical dysfunction, and the presence of serious adverse events. Serious adverse events were defined as any undesirable medical event that occurred while wearing the HAL or during the training period, or when at home, which required hospitalization for treatment or resulted in permanent or significant disability or dysfunction.

#### 2.3.2. Efficacy

Evaluations were conducted one week prior to the start of training and one week following the end of training.

##### Shoulder Joint Function

Shoulder joint ROM during voluntary and passive shoulder flexion was assessed to evaluate the shoulder joint function on the affected side, and a manual muscle test (MMT) was performed to evaluate muscle strength during shoulder flexion.

##### Surface Electromyography

Wireless surface electromyography devices were placed on the trapezius, deltoid, infraspinatus, pectoralis major, biceps brachii, and triceps brachii muscles of the impaired side, and the Trigo™ Lab wireless surface electromyography system (Delsys Inc., Boston, MA, USA) was used to evaluate muscle activity before and during HAL training, while raising the upper limb of the affected side. The obtained values were band-pass filtered (30–400 Hz), rectified and integrated over a 50 ms local time window, and divided into cycles of repeated upper limb raising exercises, after which the average activity pattern per cycle without and with HAL was obtained. We subsequently compared the activity patterns of each muscle with and without HAL at the first and tenth intervention sessions according to the peak of the averaged patterns, respectively.

##### Motion Analysis

An optical three-dimensional motion analyzer (MX System, Vicon Motion Systems Ltd., Oxford, UK) was used to analyze motion during elevation of the upper limb on the affected side. Surface markers were placed on the spinous processes of C7 and T10, at the shoulder peak and the lateral epicondyle of the humerus. The trunk axis was defined as the line from C7 to T10, and the humeral axis was defined as the line from the shoulder peak to the lateral epicondyle of the humerus. The angle between these axes was calculated and detected as the angular velocity based on the time from the drooped position to the point of maximum reach.

##### Upper Limb Function and Activity

The upper limb function on the affected side was evaluated using the sum of modified Ashworth scale (MAS) scores in the affected upper limb (range, 0–28; shoulder flexion, elbow flexion, extension forearm rotation and extraversion, and wrist flexion and extension), and Fugl Meyer assessment—upper extremity (FMA-UE), action research arm test (ARAT), and box and block test (BBT) scores. Grip strength in the sitting position was measured to indicate hand function. A digital measuring device capable of measuring at ≥5 kg was used and, if measurement was not possible, the evaluation was performed at 0 kg.

### 2.4. Statistical Analysis

All data are presented as mean ± standard deviation. A Wilcoxon signed rank test was applied to examine shoulder joint ROM, MAS scores, upper limb function scores (FMA-UE, ARAT, BBT), and surface electromyography (EMG) data in each muscle. Statistical significance was set at 5%. JMP ver. 17.0.0 software was used for all statistical analyses.

## 3. Results

### 3.1. Safety

All patients performed the 10 shoulder HAL training interventions without any apparent adverse events, including shoulder pain. The mean duration of the 10 motion training sessions was 112 ± 33.7 days (77–168 days). The average time of shoulder joint elevation per training session was 156.1 ± 31.4 min; the average time of the first training session was 93.1 ± 34.4 min, and the average time of the tenth training session was 196.3 ± 40.3 min.

### 3.2. Efficacy

#### 3.2.1. Shoulder Joint Function

**Figure 3:**
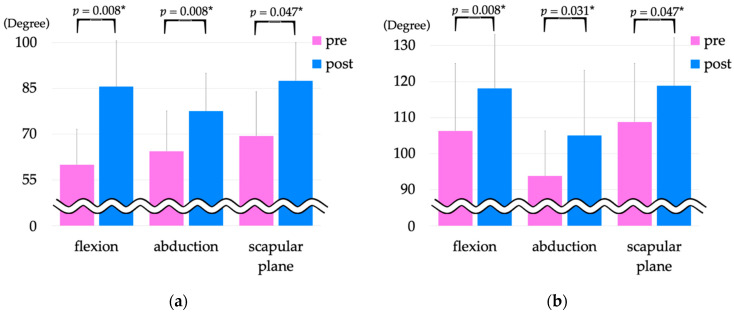
Shoulder joint ROM pre- and post-intervention. (**a**) Voluntary shoulder joint range of motion pre- and post-intervention, (**b**) passive shoulder joint ROM pre- and post-intervention. * Indicates *p* < 0.05. ROM, range of motion.

The results for shoulder joint ROM pre- and post-intervention are shown in Figure 3. Pre-intervention, voluntary shoulder joint ROM measurements were: flexion, 60.0° ± 11.6°; abduction, 64.4° ± 13.2°; and 69.4° ± 14.5° of scapular plane movement, and passive ROM measurements were: flexion, 106.3° ± 18.7°; abduction, 93.8° ± 12.5°, and 108.8° ± 16.2° of scapular plane movement. Post-intervention, voluntary shoulder joint ROM measurements were: flexion, 85.6° ± 15.0° (*p* = 0.008); abduction, 77.5° ± 12.5° (*p* = 0.008); and 87.5° ± 12.5° of scapular plane movement (*p* = 0.047). Passive shoulder joint ROM measurements were: flexion, 118.1° ± 14.9° (*p* = 0.008); abduction, 105.0° ± 18.1° (*p* = 0.031); and 118.8° ± 13.4° (*p* = 0.047) of scapular plane movement. All parameters showed significant improvement compared with pre-intervention measurements.

Pre-intervention, all eight patients had an MMT score of 2 for both flexion movements. Post-intervention, two patients showed improvement, with flexion scores improving to 4 in Patients 1 and 5.

#### 3.2.2. Surface Electromyography

**Figure 4:**
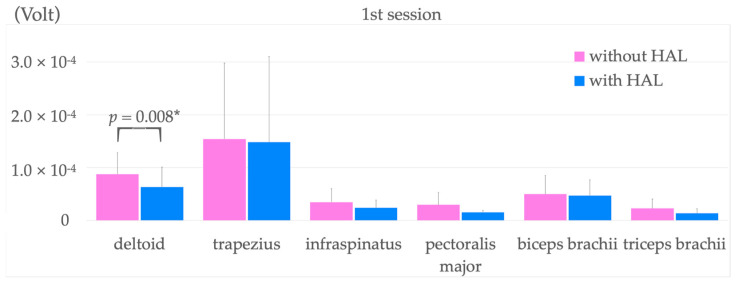
Comparison of surface EMG activity during upper limb raising before and after HAL application in the first training intervention. * *p* < 0.05. HAL, hybrid assistive limb.

The results of surface EMG before and during the initial training with and without HAL are shown in Figure 4. We compared surface EMG findings during initial upper extremity raising of the affected side with and without HAL. These showed a significant decrease in mean activity in the deltoid muscle from 8.77 × 10^−5^ ± 4.04 × 10^−5^ without HAL to 6.34 × 10^−5^ ± 3.75 × 10^−5^ when wearing HAL. No significant changes were observed in the other muscles.

**Figure 5:**
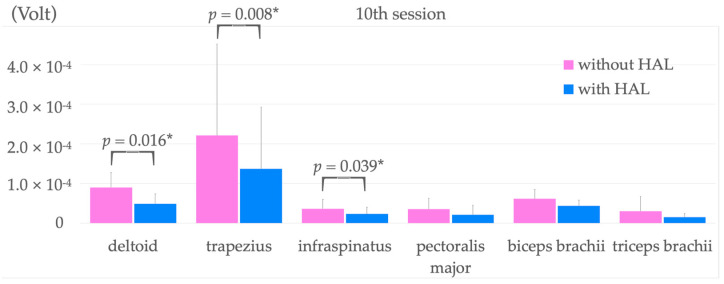
Comparison of surface EMG activity during upper limb raising before and after HAL application in the tenth training intervention session. * *p* < 0.05. HAL, hybrid assistive limb.

The surface EMG findings before and during HAL application at the tenth training session are shown in Figure 5. We compared surface EMG findings during raising the affected upper limb with and without HAL at the tenth session. These showed that mean activity significantly decreased from 8.98 × 10^−5^ ± 3.79 × 10^−5^ without HAL in the deltoid muscle to 4.88 × 10^−5^ ± 2.45 × 10^−5^ when wearing HAL (*p* = 0.016). Furthermore, a significant decrease from 2.21 × 10^−4^ ± 2.31 × 10^−4^ to 1.37 × 10^−4^ ± 1.56 × 10^−4^ was observed in the trapezius muscle (*p* = 0.008) and a decrease from 3.62 × 10^−5^ ± 2.29 × 10^−5^ to 2.29 × 10^−5^ ± 1.70 × 10^−5^ in the infraspinatus muscle (*p* = 0.039) when wearing HAL compared with not wearing HAL, respectively.

A significant decrease was observed in the deltoid muscle activity when wearing the HAL compared with prior to wearing the HAL, with no significant change observed in other muscles.

There was a significant decrease in the deltoid, trapezius, and infraspinatus muscle activity when wearing the HAL compared with prior to wearing the HAL, with no significant change noted in the other muscles.

#### 3.2.3. Motion Analysis

**Figure 6:**
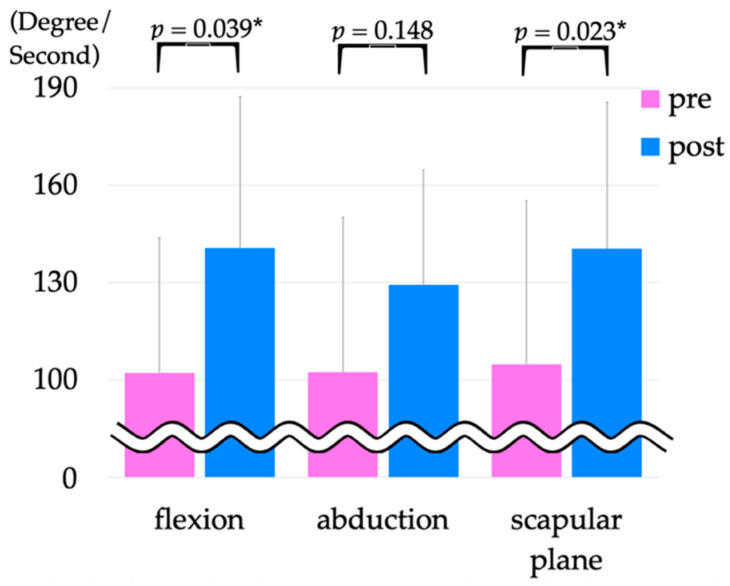
Motion analysis of raising the affected side of the upper limb in the standing position without HAL. * *p* < 0.05. HAL, hybrid assistive limb.

The maximum angular velocity during upper extremity elevation pre- and post-intervention showed a significant improvement in angle degree per second from 102.2 ± 41.6 to 140.7 ± 46.8 (*p* = 0.039) in flexion and from 104.9 ± 50.5 to 140.5 ± 45.3 (*p* = 0.023) in the scapular plane (Figure 6). We showed upper extremity elevation in Movies S1 and S2.

The maximum angular velocity during flexion and scapular elevation was significantly improved post-intervention compared with pre-intervention.

#### 3.2.4. Upper Limb Function and Activity

**Figure 7:**
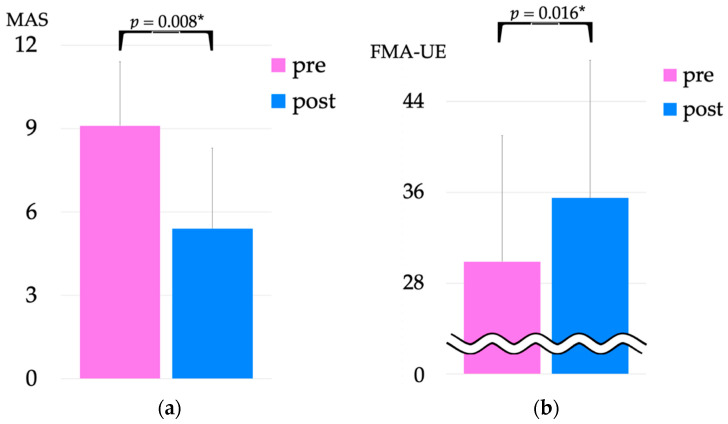
Comparison of MAS (**a**) and FMA-UE (**b**) scores pre- and post-intervention. * *p* > 0.05. FMA-UE, Fugl Meyer assessment upper extreme; MAS, modified Ashworth scale. Both MAS and FMA-UE scores significantly improved post-intervention.

The total MAS score in the upper limb showed a significant decrease from 9.1 ± 2.3 pre-intervention to 5.4 ± 2.9 post-intervention (*p* = 0.008). The results of FMA-UE test pre- and post-intervention are shown in Figure 7. The pre-intervention FMA-UE score was 29.9 ± 11.1, which significantly improved to 35.5 ± 12.1 post-intervention (*p* = 0.016).

**Figure 8:**
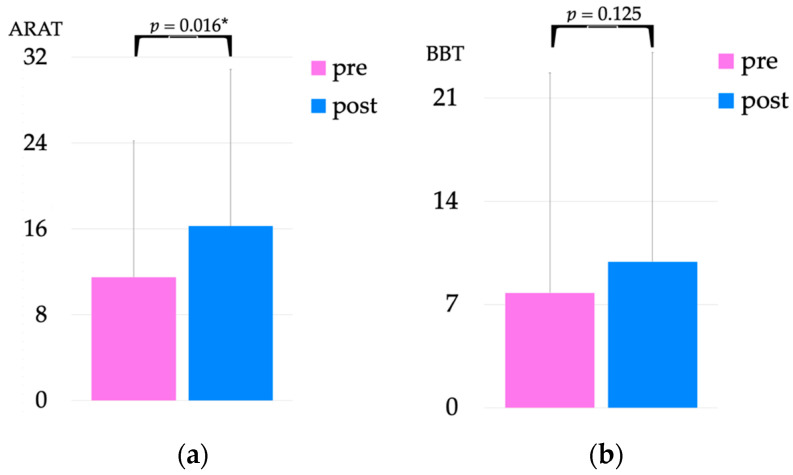
Comparison of ARAT (**a**) and BBT (**b**) scores pre- and post-intervention. * *p* < 0.05. ARAT, action research arm test; BBT, box and block test.

The results of ARAT and BBT scores pre- and post-intervention are shown in Figure 8. Pre-intervention, the ARAT score was 11.5 ± 12.7, which significantly improved to 16.25 ± 14.6 post-intervention (*p* = 0.016). Pre-intervention, the BBT score was 7.8 ± 14.9, which improved to 9.9 ± 14.2 post-intervention but without statistical significance (**p* =* 0.125). Three of eight patients had hand function difficulties, and their BBT scores were 0 both pre- and post-intervention.

Post-intervention grip strength did not improve significantly; however, two of the three patients who had pre-intervention difficulty achieving a measurement could achieve a post-intervention measurement, with Patient 1 improving grip strength to 7.4 kg and Patient 4 improving grip strength to 9 kg.

## 4. Discussion

In this study, we aimed to test the safety and effectiveness of a shoulder joint HAL through using HAL to treat patients with stroke and moderate-to-severe upper limb dysfunction. In all eight patients, we observed shoulder joint contracture and muscle weakness on the affected side but training the upper limb to rise using the shoulder joint HAL could be performed safely without shoulder pain. Due to its instability, the shoulder joint is prone to shoulder pain when an upper limb is affected and, once hemiplegic shoulder joint pain develops, it is challenging to resolve and can significantly affect patient’s quality of life [2,13]. Therefore, in conventional training, avoiding shoulder flexion beyond 90° is recommended to prevent shoulder pain [14,15]. Our study findings indicate that this technique can be safely performed if the shoulder MMT score is maintained at a muscle strength of at least 2, even if a relatively severe limitation in shoulder joint ROM is observed.

Limb dysfunction after stroke is problematic, not only in terms of muscle weakness, but also in terms of coordinated muscle movements. HAL treatment has the possibility of improving coordination through motor learning [16]. We have also used HAL for elbow extension training for patients with spastic cerebral palsy, with a focus on achieving elbow flexor and extensor movement separately. Coactivity between biceps and triceps brachii decreased following HAL sessions and active elbow extension improved [17]. Previously, we published reports showing improved coordinated movement in a patient with chronic stroke and in patients after C5 palsy [9,18,19].

In this study concerning evaluation of shoulder joint ROM, surface EMG played a central role in motion analysis. The shoulder joint has a high degree of freedom, accompanied with various types of muscle movement. Previous studies have utilized different methods to analyze shoulder movements. Tigrini et al. evaluated motion intention through pattern recognition methods in relation to upper limb surface EMG [20,21]. Rivela et al. evaluated the surface EMG of trunk muscles other than upper limb muscles [22,23]. Additionally, several studies examined patients with shoulder disarticulation, focusing on the shoulder joint itself [23,24,25]. In our study, we selected the trunk muscles, including the trapezius, deltoid, infraspinatus, and pectoralis major muscles as well as upper limb muscles, such as biceps brachii and triceps brachii muscles, to evaluate surface EMG with and without HAL, based on previous studies [17,18,26].

Surface EMG findings showed co-activation of the trapezius, infraspinatus, and deltoid muscles when the upper limb was raised without the HAL in both the first and tenth sessions. In contrast, during the initial upper limb raising with HAL, only the deltoid muscles showed a decrease in activation. However, during the tenth training session, a significant decrease in activation was observed in the trapezius and infraspinatus muscles compared with upper limb raising without HAL, suggesting reduced co-activation of these muscles during upper limb raising with HAL. In a previous report [26], in which healthy participants performed upper limb raising with HAL, there was more contraction of the deltoid, trapezius, and infraspinatus muscles with HAL than without. These results suggest that HAL treatment for the shoulder joint in the present study improved muscle coordination during upper limb raising, and that treatment using the shoulder HAL for patients with chronic stroke and upper limb dysfunction may have the same motor learning effect as shown in previous reports [9,16,17,18,19]. Post-intervention, our study patients also showed improvement in muscle tone of the entire upper extremity, which we consider also contributed to improvement in coordinated movement.

To evaluate efficacy, patients in the chronic phase six months after stroke onset were included to exclude recovery of function and movement due to natural progression after stroke [27]. After shoulder HAL intervention, both voluntary and passive shoulder joint ROM significantly improved, and motion analysis showed that the patients were able to raise the affected upper extremity higher and more quickly, suggesting improved peri-shoulder muscle strength. The MMT is only a reference evaluation, as it is affected by automatic ROM as well as by muscle strength; however, two of eight patients showed improvement (from 2 to 4), which suggested an improvement in muscle strength. Tests to assess upper extremity function and movement, namely, the FMA and ARAT, also showed significant score improvement post-intervention, which indicates that there was improvement in the upper limb as a whole, along with improved shoulder joint function. Moreover, two of eight patients showed improvements in grip strength, which they had been unable to perform pre-intervention, indicating an improvement in hand function. Overall, our results show that shoulder HAL is a safe and effective treatment for patients with chronic stroke and moderate-to-severe upper limb dysfunction.

### Limitations

The limitations of this study are as follows. First, to be cautious and to closely monitor the development of shoulder pain, this intervention was performed 10 times with at least one week between each intervention. Therefore, the 10 interventions were completed in an average of 112 days, resulting in a low intervention frequency. Second, the intervention targeted patients in the chronic phase to remove the influence of spontaneous recovery after stroke and an average of 5.86 years had passed from stroke onset to intervention. Third, the study environment was not conducive to effective rehabilitation for patients with stroke, as rehabilitation after stroke is more effective when undertaken at a shorter time from stroke onset and the amount of rehabilitation is more effective when performed 5–7 days per week [28]. Therefore, it is necessary to consider when and how to perform shoulder HAL treatment in future, as training may be more effective if intervention studies are conducted earlier and more frequently. Finally, when selecting our study patients, we focused on shoulder joint function only. As such, pre-intervention grip strength measurements could not be achieved in three of eight patients and hand function was often sub-optimal in the other patients. Upper limb movement is effective only when the patient can perform “grip and release” and “pinch and release” hand movements, in addition to reaching movements of the shoulder and elbow joints, and ARAT and BBT evaluations are based on this assumption [29,30]. Upper limb dysfunction after stroke varies from patient to patient, and it is important to decide which training should be administered to which patients [31]. Our study results suggest that HAL can improve shoulder joint function and also improve upper limb function and movement.

## 5. Conclusions

We used shoulder joint elevation training using a single-joint HAL in eight patients with chronic stroke and moderate-to-severe upper limb dysfunction. Shoulder joint ROM improved, suggesting an increase in muscle output of the peri-articular muscles of the shoulder joint. In addition, improvements in muscle tone of the entire upper limb were observed, and significant improvements in FMA-UE and ARAT scores were also obtained, indicating improvements in function and movement of the affected upper limb. These findings suggest that the shoulder HAL may be an effective rehabilitation strategy for upper limb dysfunction after stroke.

## Figures and Tables

**Table 1:** Patient characteristics.

Number	Age	Sex	Disease	Side	From Onset (Year)	Shoulder MMT		Grip Strength (kg)	
						Affected Side	Unaffected Side	Affected Side	Unaffected Side
1	53	Female	SAH	Rt	1.98	2	5	0	17.8
2	84	Male	ICH	Rt	8.24	2	5	8.1	31.3
3	67	Male	CI	Lt	0.93	2	5	9.7	26.3
4	68	Male	ICH	Rt	8.65	2	5	0	33.4
5	71	Male	CI	Lt	1.60	2	5	6.7	30.3
6	68	Male	CI	Lt	4.57	2	5	15.7	35.1
7	67	Male	ICH	Lt	1.20	2	5	11	27.5
8	69	Female	CI	Lt	19.7	2	5	0	-

CI, cerebral infarction; ICH, intracranial hemorrhage; Lt, left; MMT, manual muscle testing; Rt, right; SAH, subarachnoid hemorrhage.

## Data Availability

Not applicable.

## Supplementary Materials

The following supporting information can be downloaded at: https://www.mdpi.com/article/10.3390/jcm12031215/s1. Movie S1. Elevation of the affected side of the upper limb with shoulder flexion pre-intervention in Patient 5. Movie S2. Elevation of the affected side of the upper limb with shoulder flexion post-intervention in Patient 5.

## References

[1] Lloyd-Jones D., Adams R.J., Brown T.M., Carnethon M., Dai S., De Simone G., Ferguson T.B., Ford E., Furie K., Gillespie C. (2010). Executive summary: Heart disease and stroke statistics—2010 update: A report from the American Heart Association. Circulation.

[2] Broeks J.G., Lankhorst G.J., Rumping K., Prevo A.J. (1999). The long-term outcome of arm function after stroke: Results of a follow-up study. Disabil. Rehabil..

[3] Lai C.H., Sung W.H., Chiang S.L., Lu L.H., Lin C.H., Tung Y.C., Lin C.H. (2019). Bimanual coordination deficits in hands following stroke and their relationship with motor and functional performance. J. Neuroeng. Rehabil..

[4] Kwakkel G., Kollen B.J., van der Grond J., Prevo A.J. (2003). Probability of regaining dexterity in the flaccid upper limb: Impact of severity of paresis and time since onset in acute stroke. Stroke.

[5] Marshall R.S., Perera G.M., Lazar R.M., Krakauer J.W., Constantine R.C., Delapaz R.L. (2000). Evolution of cortical activation during recovery from corticospinal tract infarction. Stroke.

[6] Bertani R., Melegari C., De Cola M.C., Bramanti A., Bramanti P., Calabrò R.S. (2017). Effects of robot-assisted upper limb rehabilitation in stroke patients: A systematic review with meta-analysis. Neurol. Sci..

[7] Saita K., Morishita T., Arima H., Hyakutake K., Ogata T., Yagi K., Shiota E., Inoue T. (2018). Biofeedback effect of hybrid assistive limb in stroke rehabilitation: A proof of concept study using functional near infrared spectroscopy. PLoS ONE.

[8] Okuno T., Takeuchi T., Takeda E., Izumi Y., Kaji R. (2021). Clinical uses of a robot (hybrid-assisted limb or HAL™) in patients with post-stroke spasticity after botulinum toxin injections. J. Med. Investig..

[9] Taketomi M., Shimizu Y., Kadone H., Hada Y., Yamazaki M. (2021). Hybrid Assistive Limb Intervention for Hemiplegic Shoulder Dysfunction Due to Stroke. Cureus.

[10] Kamkar A., Irrgang J.J., Whitney S.L. (1993). Nonoperative management of secondary shoulder impingement syndrome. J. Orthop. Sports Phys. Ther..

[11] Lindgren I., Lexell J., Jönsson A.C., Brogårdh C. (2012). Left-sided hemiparesis, pain frequency, and decreased passive shoulder range of abduction are predictors of long-lasting poststroke shoulder pain. PMR.

[12] Olczak A., Truszczyńska-Baszak A. (2021). Influence of the passive stabilization of the trunk and upper limb on selected parameters of the hand motor coordination, grip strength and muscle tension, in post-stroke patients. J. Clin. Med..

[13] Pong Y.P., Wang L.Y., Wang L., Leong C.P., Huang Y.C., Chen Y.K. (2009). Sonography of the shoulder in hemiplegic patients undergoing rehabilitation after a recent stroke. J. Clin. Ultrasound.

[14] Ludewig P.M., Cook T.M. (2000). Alterations in shoulder kinematics and associated muscle activity in people with symptoms of shoulder impingement. Phys. Ther..

[15] Patel R.M., Gelber J.D., Schickendantz M.S. (2018). The weight-bearing shoulder. J. Am. Acad. Orthop. Surg..

[16] Puentes S., Kadone H., Watanabe H., Ueno T., Yamazaki M., Sankai Y., Marushima A., Suzuki K. (2018). Reshaping of bilateral gait coordination in hemiparetic stroke patients after early robotic intervention. Front. Neurosci..

[17] Shimizu Y., Kadone H., Kubota S., Ueno T., Sankai Y., Hada Y., Yamazaki M. (2019). Voluntary elbow extension-flexion using single joint Hybrid Assistive Limb (HAL) for patients with spastic cerebral palsy: Two case reports. Front. Neurol..

[18] Kubota S., Kadone H., Shimizu Y., Takahashi H., Koda M., Miura K., Watanabe H., Suzuki K., Hada Y., Sankai Y. (2021). Robotic shoulder rehabilitation with the hybrid assistive limb in a patient with delayed recovery after postoperative C5 palsy: A case report. Front. Neurol..

[19] Lafitte M.N., Kadone H., Kubota S., Shimizu Y., Tan C.K., Koda M., Hada Y., Sankai Y., Suzuki K., Yamazaki M. (2022). Alteration of muscle activity during voluntary rehabilitation training with single-joint Hybrid Assistive Limb (HAL) in patients with shoulder elevation dysfunction from cervical origin. Front. Neurosci..

[20] Tigrini A., Pettinari L.A., Verdini F., Fioretti S., Mengarelli A. (2021). Shoulder Motion Intention Detection Through Myoelectric Pattern Recognition. IEEE Sens. Lett..

[21] Tigrini A., Scattolini M., Mengarelli A., Fioretti S., Morettini M., Burattini L., Verdini F. Role of the Window Length for Myoelectric Pattern Recognition in Detecting User Intent of Motion. Proceedings of the 2022 IEEE International Symposium on Medical Measurements and Applications (MeMeA).

[22] Rivela D., Scannella A., Pavan E.E., Frigo C.A., Belluco P., Gini G. (2018). Analysis and Comparison of Features and Algorithms to Classify Shoulder Movements from sEMG Signals. IEEE Sens. J..

[23] Rivela D., Scannella A., Pavan E.E., Frigo C.A., Belluco P., Gini G. Processing of surface EMG through pattern recognition techniques aimed at classifying shoulder joint movements. Proceedings of the 2015 37th Annual International Conference of the IEEE Engineering in Medicine and Biology Society (EMBC).

[24] Sharba G.K., Wali M.K., Ai-Timemy A.H. (2019). Real-time classification of shoulder girdle motions for multifunctional prosthetic hand control: A preliminary study. Int. J. Artif. Organs.

[25] Nsugbe E., Al-Timemy A.H. (2022). Shoulder girdle recognition using electrophysiological and low frequency anatomical contraction signals for prosthesis control. CAAI Trans. Intell. Technol..

[26] Makihara T., Kadone H., Onishi S., Kubota S., Hada Y., Shimizu Y., Kawamoto H., Sankai Y., Yamazaki M. (2017). Shoulder motion assistance using a single-joint Hybrid Assistive Limb^®^ robot: Evaluation of its safety and validity in healthy adults. J. Orthop. Surg..

[27] Nakayama H., Jørgensen H.S., Raaschou H.O., Olsen T.S. (1994). Recovery of upper extremity function in stroke patients: The Copenhagen Stroke Study. Arch. Phys. Med. Rehabil..

[28] Pollock A., Baer G., Campbell P., Choo P.L., Forster A., Morris J., Pomeroy V.M., Langhorne P. (2014). Physical rehabilitation approaches for the recovery of function and mobility following stroke. Cochrane Database Syst. Rev..

[29] Hsieh C.L., Hsueh I.P., Chiang F.M., Lin P.H. (1998). Inter-rater reliability and validity of the action research arm test in stroke patients. Age Ageing.

[30] Slota G.P., Enders L.R., Seo N.J. (2014). Improvement of hand function using different surfaces and identification of difficult movement post stroke in the Box and Block Test. Appl. Ergon..

[31] Jolkkonen J., Kwakkel G. (2016). Translational hurdles in stroke recovery studies. Transl. Stroke Res..

